# Effects of Cardiomyopathic Mutations on the Cytoplasmic Tropomyosin Isoform Tpm1.7

**DOI:** 10.3390/molecules31111784

**Published:** 2026-05-22

**Authors:** Svetlana G. Roman, Salavat R. Nabiev, Anastasia M. Kochurova, Galina V. Kopylova, Julia Y. Antonets, Sergey Y. Kleymenov, Valeriya V. Mikhaylova, Daniil V. Shchepkin, Alexander M. Matyushenko, Victoria V. Nefedova

**Affiliations:** 1 Research Centre of Biotechnology, Russian Academy of Sciences, 119071 Moscow, Russia; svetabaj@gmail.com (S.G.R.); s.yu.kleymenov@gmail.com (S.Y.K.); mikhaylova.inbi@inbox.ru (V.V.M.); ammatyushenko@mail.ru (A.M.M.); 2 Institute of Immunology and Physiology, Ural Branch of Russian Academy of Sciences, 620049 Yekaterinburg, Russia; salavatik2003@gmail.com (S.R.N.); uliaantonec@gmail.com (J.Y.A.); cmybp@mail.ru (D.V.S.); 3 Koltzov Institute of Developmental Biology, Russian Academy of Sciences, 119334 Moscow, Russia

**Keywords:** actin filaments, actin cytoskeleton dynamics, cytoplasmic isoforms of tropomyosin, optical trap, differential scanning calorimetry, cardiomyopathies

## Abstract

Tropomyosins (Tpm) are the family of actin-binding proteins encoded by four genes in humans. Missense mutations in the *TPM1* gene associated with cardiomyopathies have been studied in the sarcomeric isoform Tpm1.1. The cardiomyopathy-causing mutations E40K and E54K are located in exon 2b of the *TPM1* gene and may be expressed in non-muscle cytoplasmic Tpm isoforms, including Tpm1.7, which is associated with early tissue development. In the present work, we investigate the effects of mutations E40K and E54K on the properties of Tpm1.7. The E40K and E54K mutations caused destabilization of the Tpm1.7 molecule at the N- and C-termini parts. Neither mutation affected the Tpm1.7 affinity for filamentous actin (F-actin). The bending stiffness of F-actin/Tpm1.7 E40K filaments was lower compared to F-actin/Tpm1.7 WT (wild-type). The interplay of Tpm1.7 and motor proteins was studied in an in vitro motility assay with skeletal myosin. Tpm1.7 WT reduced the sliding velocity of F-actin by half; the velocity of F-actin with Tpm1.7 E54K did not differ from that of bare F-actin; and Tpm1.7 E40K decreased the F-actin velocity by approximately threefold. While Tpm1.7 E40K did not affect the protective effect of Tpm1.7 against F-actin severing by cofilin-1, the E54K mutation enhanced protection against cofilin-1. Thus, cardiomyopathic mutations in the *TPM1* gene can affect the properties of non-muscle Tpm isoforms, which indicates that this should be taken into account when studying the molecular mechanisms of the pathogenesis of these diseases.

## 1. Introduction

Tropomyosins (Tpm) constitute a family of coiled-coil proteins that interact with filamentous actin (F-actin) [1]. Around 30 isoforms of Tpm have been described for human tissues to date. Such diversity is due to four Tpm encoded genes (*TPM1-TPM4*), alternative splicing, and alternative promoters [2]. All Tpm molecules display common features, such as a dimeric coiled-coil structure, heptad repeats in the amino acid (a.a.) sequence, self-assembly through N-to-C ends (the so-called overlap region) interactions, and an ability to form prolonged polymers on actin filaments [1,3]. The a.a. in heptads are designated as (*a,b,c,d,e,f,g*)_n_ and reflect the position of an a.a. in the coiled-coil structure. The a.a. at *a* and *d* positions are located in the core and often contain hydrophobic a.a. residues (such as Leu, Ile, Val). The a.a. at *e* and *g* positions interact electrostatically [3]. The human *TPM1* gene contains ten alternatively spliced exons (1a, 2a, 2b, 1b, 6a, 6b, 9a–d) and five constitutive exons (3,4,5,7,8) and expresses ten Tpm isoforms that differ in their tissue specificity and properties [2].

Missense mutations in the *TPM1* gene encoding Tpm1.1 associate with the inherited cardiac diseases, cardiomyopathies [4,5]. Mutations E40K (ClinVar NM_001018005.2(TPM1):c.118G>A (p.Glu40Lys) and E54K (ClinVar NM_001018005.2(TPM1):c.160G>A (p.Glu54Lys) in *TPM1* are known to be associated with dilated cardiomyopathy [6]. Since Tpm1.1 is the major protein isoform in cardiomyocyte sarcomeres, the effects of these mutations on this isoform have been well studied. In thin filaments, this isoform, together with troponin, regulates muscle contraction and the interaction of myosin with actin in the presence of Ca^2+^ [7]. It has been previously shown that E40K mutation reduces Ca^2+^ sensitivity of thin filaments in both ATPase activity and in vitro motility assay [8,9]. In the case of the E54K mutation, some studies found a decrease in calcium sensitivity [8], while in other research it did not change [9]. The E40K mutation reduced the affinity of Tpm1.1 for actin by 1.7-fold in the «On» state (in the presence of S1 myosin), whereas the E54K mutation reduced the affinity by 3.5-fold in the «Off» state. Both mutations are located at position *e* in the heptad and result in strong destabilization of the N-terminus [8]. Engineered heart tissues (EHTs) with cardiomyocytes carrying the *TPM1* E54K mutation exhibited an almost 3-fold decrease in the active peak force compared to cardiomyocytes carrying a Tpm1 WT, a shorter twitch and a shorter time to reach peak force than for WT EHTs, and took only half as much time as WT EHTs to relax [10]. Both mutants affected the pCa-tension dependence, indicating that contractility was decreased during systole and over-inhibited during diastole [11].

**Figure 1:**
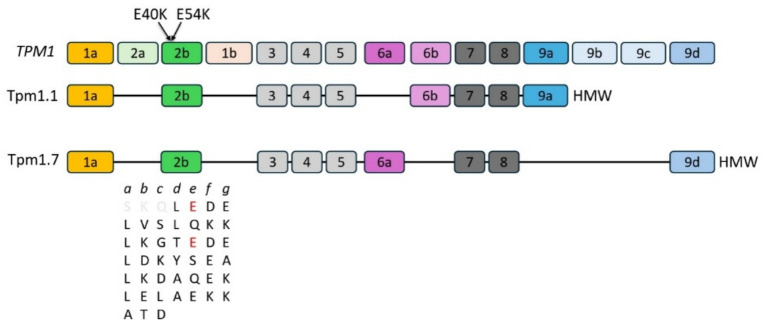
The exon composition of Tpm1.1 and Tpm1.7 isoforms of the human *TPM1* gene. The positions of E40K and E54K mutations in exon 2b are marked by arrows.

The E40K and E54K mutations are located in exon 2b of the *TPM1* gene and, therefore, they may be in all Tpm isoforms containing this exon, such as Tpm1.5, Tpm1.6, and Tpm1.7 [2]. Here, we have studied the effects of these mutations in the Tpm1.7 isoform (previously known as Tm3). Unlike Tpm1.1, this isoform has a different central exon 6 and a C-terminal exon 9 (Figure 1). Due to exon 9d, Tpm1.7 forms strong head-to-tail interactions between adjacent molecules, has one of the highest viscosities among Tpm isoforms [12], and a medium affinity to F-actin compared to other Tpm isoforms [13].

The Tpm1.7 isoform belongs to HMW (high molecular weight) isoforms and is usually found in embryonic brain tissues, cleavage furrow, filopodia, and stress fibers [14,15]. Tpm1.7 was also found in a specific type of contractile stress fibers, which include the contractile actin rings that pinch the cell membrane in cytokinesis and support cell to cell adhesions in epithelial cells [15]. Despite Tpm1.7 not being expressed in adult muscles [16], its ectopic expression in mice resulted in Tpm1.7 appearance in the Z-line adjacent structures of muscles [17]. Such transgenic mice exhibited muscular dystrophy and ragged-red fiber phenotype, indicating a disruption of the integrity of the membrane-associated cytoskeleton [17].

The Tpm expression patterns differ not only among the cell types, but also within one cell. Sub-populations of functionally distinct actin filaments can be formed in cells through Tpm isoforms [18]. Overexpression of Tpm1.7 causes filopodia formation by recruiting actin depolymerizing factors [19].

Cytoskeleton dynamics should maintain a certain speed, and any deviations (either acceleration or slowing down) can lead to a pathogenic phenotype. Cofilin activity is one of the key regulators of actin filaments dynamics (the rates of severing and polymerization/depolymerization) [20,21,22,23]. Taking into account that most of F-actin in cells is present in the form of actin-tropomyosin co-polymers [24], Tpms could affect various actin-related processes, as well as cofilin activity [23]. ERK1/2-mediated cofilin activation has also been shown to be involved in cardiomyopathy development [25,26,27]. Tpm1.7 has been shown to stably interact with actin filaments and protect them from ADF/cofilin-mediated disassembly [28]. The mechanism of that is not yet fully understood, but Tpm1.7 was shown to significantly inhibit binding of cofilin-1 to the actin filaments [29]. It is worth considering that Tpm homeostasis in the cell is based on the balance between its degradation in proteasomes and de novo synthesis. For HMW Tpm isoforms (including Tpm1.7, Tpm1.6, and Tpm2.1), it has been shown that their proteasomal turnover depends on their dissociation from actin filaments [30].

In the present study, we assessed the effects of E40K and E54K mutations in Tpm1.7 isoform on the structural properties of the Tpm molecule and Tpm binding to actin filaments. We also examined the interactions of F-actin decorated with Tpm1.7 with such actin-binding proteins as cofilin-1 and myosin. We assume that overall, these data may shed light on the Tpm1.7 activity in normal and pathogenic cells.

## 2. Results

### 2.1. Structural Properties of Tpm1.7 E40K and E54K Variants

**Figure 2:**
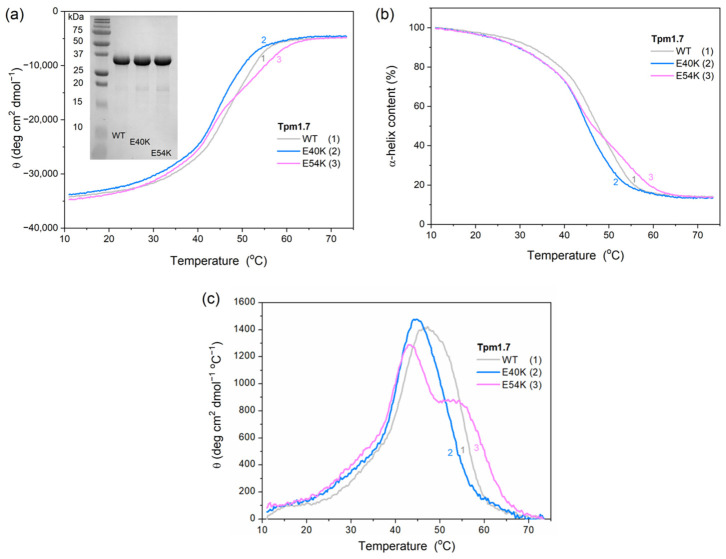
Thermal stability of Tpm1.7 WT, Tpm1.7 E40K, and Tpm1.7 E54K measured by circular dichroism (CD). (**a**) Representative CD melting curves of Tpms recorded at 222 nm. The SDS-PAGE represents the purity of protein preparations. (**b**) Content of α-helical structures in Tpms. (**c**) The first derivative analysis of the CD melting curves presented in panel (**a**).

To study the structure of Tpm1.7 E40K and E54K mutants, we applied thermal stability assays. Since Tpm1.7 is a coiled-coil protein, we first performed circular dichroism measurements and detected heat-induced changes in the molar ellipticity (Figure 2a,b). The first derivative analysis was used to identify thermal transitions in the melting curves (Figure 2c). Tpm1.7 WT exhibited one major asymmetrical peak at 47 °C and a low-temperature shoulder. The E40K mutation resulted in an overall shift of the melting curve toward lower temperatures, with the major peak at ~42 °C. The melting curve shape of the Tpm1.7 E54K mutant displayed both a new low-temperature major peak at ~41 °C and a new high-temperature peak at ~55 °C.

**Figure 3:**
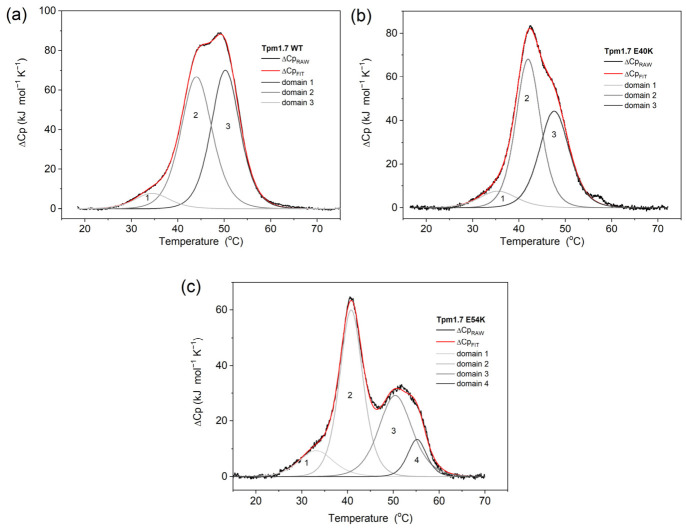
Temperature dependences of the excess heat capacity (ΔC_p_) of Tpm1.7 WT (**a**), Tpm1.7 E40K (**b**), and Tpm1.7 E54K (**c**) obtained by differential scanning calorimenty (DSC). Representative experimental DSC curves after subtracting instrumental and chemical baselines are shown by solid black lines, solid red lines are obtained by fitting the data to the non-two-state model, and grey lines represent the calorimetric domains (individual thermal transitions) obtained by the deconvolution analysis. The calculated values of T_m_ and ΔH_cal_ are presented in Table 1.

The differential scanning calorimetry (DSC) with computer-assisted deconvolution analysis allows identification of the exact temperatures of thermal transition as well as their enthalpy. The DSC results (shown in Figure 3 and Table 1) demonstrated that Tpm1.7 WT had three structural parts that melted independently (so-called, calorimetric domains) at 35.2, 44.2, and 51.4 °C. The Tpm1.7 E40K also contained three calorimetric domains with T_m_ at 35.5, 41.9, and 47.6 °C. In contrast, Tpm1.7 E54K melted at four thermal transitions with T_m_ 32.8, 40.8, 50.5, and 55.2 °C. This mutation caused both a decrease in the T_m_ value of the three calorimetric domains and an appearance of a new domain at higher temperatures. Previously, it has been shown that Tpm1.1 E54K mutant also contains four calorimetric domains [8]. Thus, this phenomenon is likely a result of the amino acid substitution rather than of the Tpm isoform.

### 2.2. N-to-C Interactions in Tpm1.7 E40K and E54K Variants

Tpm molecules assemble longitudinally by overlapping their N- and C-regions. This property is reflected in the viscosity of Tpm solutions and can be estimated by viscosimetry (Table 2). Both mutant variants of the protein had excess solution viscosity similar to that of Tpm1.7 WT; therefore, the mutations did not affect the end-to-end interaction of Tpm molecules.

### 2.3. Tpm1.7 E40K and E54K Interaction with Filamentous Actin

**Figure 4:**
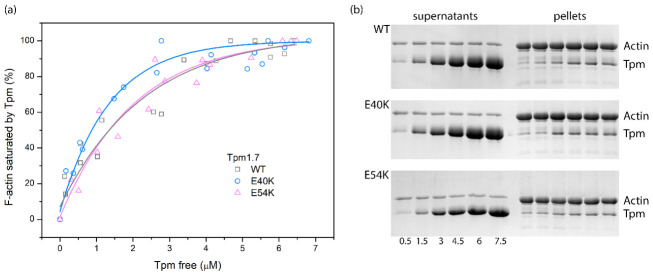
The affinity of Tpm1.7 WT, Tpm1.7 E40K, and Tpm1.7 E54K for F-actin estimated by co-sedimentation at 100,000× *g*. The experiment was performed in a 30 mM HEPES-NaOH buffer (pH 7.3), with 200 mM NaCl. (**a**) The fractions of Tpm associated with F-actin were plotted against the concentration of unbound Tpm in the supernatant. (**b**) The representative SDS-PAGEs of cosedimentation of F-actin with Tpm1.7 WT, Tpm1.7 E40K, and Tpm1.7 E54K. The total concentrations of Tpm in samples are presented under the gels. The k_50%_ (presented as mean ± SD) were 1.3 ± 0.3 µM for Tpm1.7 WT, 0.8 ± 0.1 µM for Tpm1.7 E40K, and 1.3 ± 0.4 µM for Tpm1.7 E54K. There is no statistically significant difference between the k_50%_ for Tpm1.7 E40K or Tpm1.7 E54K, and Tpm1.7 WT. Statistical differences were assessed using the nonparametric Mann–Whitney U test at a significance level of *p* < 0.05.

F-actin is the major protein partner of Tpms in cells; therefore, we studied the affinity of Tpm1.7 variants for F-actin by a co-sedimentation assay with the skeletal actin (Figure 4). Janco et al. have previously demonstrated that the affinity of Tpm1.7 does not depend on the actin isoform, and remains the same with both skeletal and cytoplasmic actin [13]. The k_50%_ (the concentration of the free Tpm of the half-saturated F-actin) for Tpm1.7 WT was 1.3 ± 0.3 µM. The k_50%_ of Tpm1.7 E40K and E54K variants were similar to Tpm1.7 WT, at 0.8 ± 0.1 µM and 1.3 ± 0.4 µM, respectively.

To assess the mechanical properties of actin filaments with Tpm1.7 WT and its mutants we measured the bending stiffness. Bare F-actin or F-actin coated by Tpm1.7 variants was stretched in a two-beam optical trap, while the force and distance between the two beads were registered. The results are shown in Table 3. The Tpm1.7 isoform statistically significantly increased the stiffness of F-actin. The Tpm1.7 E40K and E54K mutants also increased the bending stiffness of actin filaments. However, the effect of Tpm1.7 E40K was less pronounced than that of Tpm1.7 E54K.

### 2.4. Cofilin-1 Ability to Sever F-Actin Decorated with Tpm1.7 E40K and E54K Variants

**Figure 5:**
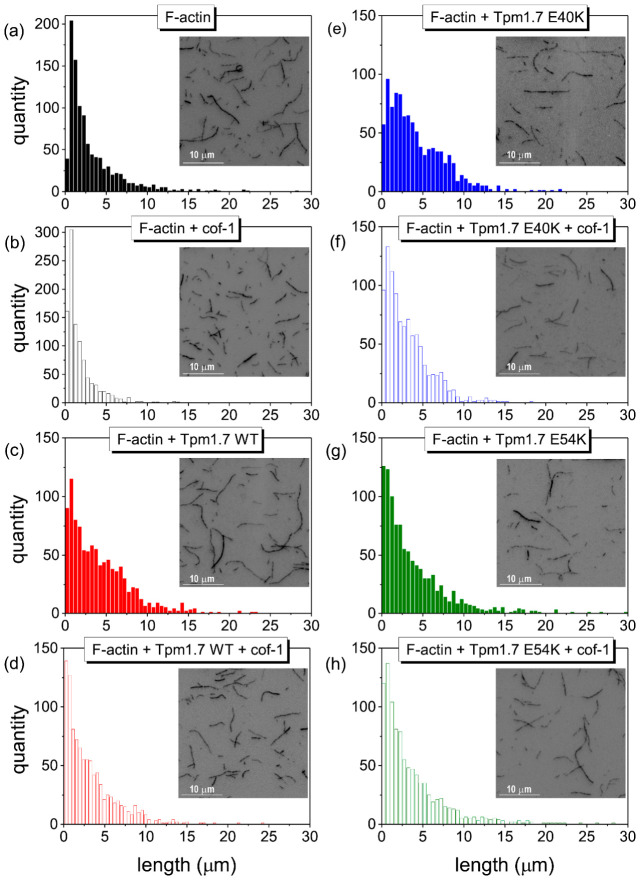
F-actin severing in the presence of cofilin-1 (cof-1). Filament length distributions of (**a**) bare F-actin; (**b**) F-actin with cof-1; (**c**) F-actin with Tpm1.7; (**d**) F-actin with Tpm1.7 and cof-1; (**e**) F-actin with Tpm1.7 E40K; (**f**) F-actin with Tpm1.7 E40K and cof-1; (**g**) F-actin with Tpm1.7 E54K; (**h**) F-actin with Tpm1.7 E54K and cof-1. Fluorescence micrographs of TRITC-rhodamine phalloidin stained filaments used for quantification. The histograms for distribution of length of filaments were obtained from 1000 filaments for each sample. Bar is 10 µm. To determine the significance of the differences, Kolmogorov–Smirnov test was applied. All samples, except F-actin/Tpm1.7 E54K and F-actin/Tpm1.7 E54K/cof-1 pair, significantly differed from each other, with *p* ≤ 0.01.

In cells, the actin dynamics is regulated by many actin-binding proteins. We studied the ability of cofilin-1 (cof-1) to sever F-actin covered by Tpm1.7 mutated variants. In order to do this, we measured the lengths of the actin filaments (stained with TRITC-rhodamine phalloidin) using fluorescence microscopy (Figure 5). Binding of cof-1 to F-actin leads to the rhodamine-phalloidin decay from filaments [31]; therefore, we stained F-actin after its incubation with cof-1. The chosen concentrations permitted simultaneous binding of both cofilin and phalloidin to F-actin, ensuring uniform filament staining suitable for microscopy. The reagent addition sequence also minimized fluorescence bleaching and prevented potential variability in cofilin activity between Tpm1.7-decorated and bare F-actin. The lengths of F-actin/Tpm1.7 filaments statistically significantly differed from those of bare F-actin with a tendency of filament length to increase in the presence of Tpm1.7 (Figure 5a vs. Figure 5c). Cof-1 exhibited its severing/disassembling activity and resulted in the formation of the population of filaments shorter than 1 µm (Figure 5 b). Actin filaments saturated with Tpm1.7 WT (Figure 5d) were significantly resistant to the severing/depolymerizing effect of cof-1. Both Tpm1.7 E40K and Tpm1.7 E54K protected actin filaments from severing (Figure 5e–h), nevertheless, the effect of Tpm1.7 E54K was more pronounced. The distribution of F-actin length with or without cof-1 for samples containing Tpm1.7 E54K did not differ from each other (Figure 5g vs. Figure 5h).

**Figure 6:**
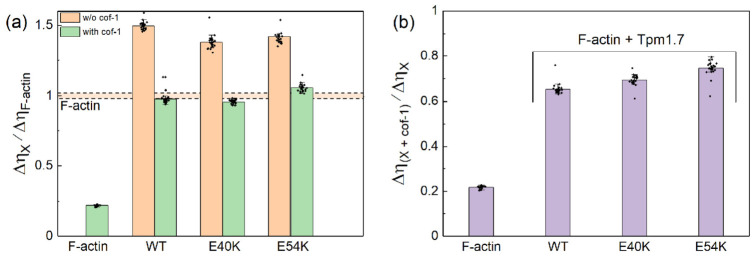
The effect of cof-1 on the viscosity of the solutions of actin filaments decorated with Tpm1.7 WT, Tpm1.7 E40K, and Tpm1.7 E54K. All values are presented as the mean ± 95% confidence interval. (**a**) The ratio of the excess viscosity (Δη_X_) of F-actin solutions with different Tpm1.7 variants (orange) and/or Tpm1.7 variants with cof-1 (green) to the excess viscosity of bare F-actin (Δη_F-actin_). (**b**) The ratio of the excess viscosity of F-actin solutions with or without Tpm1.7 variants in the presence of cof-1 (Δη_X+cof-1_) to the viscosity of the corresponding solutions in the absence of cof-1 (Δη_X_). To determine the significance of the differences in the viscosities, Student’s *t*-test was used (n = 24). The viscosity of the samples F-actin ± cofilin, F-actin ± Tpm1.7, F-actin + cofilin ± Tpm, differed significantly (*p* < 0.05).

The length of actin filaments correlated with the viscosity of solutions (Figure 6). As can be seen from Figure 5, F-actin decorated with Tpm1.7 formed significantly longer filaments compared to bare F-actin (Figure 5a,c,e,g). The excess viscosity (Δη) of the corresponding solutions also differed significantly—by 1.3–1.5-times (Figure 6a). Addition of cof-1 appreciably reduced the lengths of F-actin and the viscosity of its solution (Figure 5b and Figure 6a). The viscosity of the samples containing F-actin/Tpm1.7/cof-1 was five times higher than the viscosity of the bare F-actin/cof-1 sample. Thus, here we can also observe the protective effect of Tpm1.7 against cof-1 activity. To compare the properties of Tpm1.7 mutant variants, we assessed the ratio of the excess viscosity of F-actin solutions in the presence of different Tpm1.7 variants and cof-1 (Δη_F-actin+Tpm1.7+cof-1_) to the viscosity of the corresponding solutions without cof-1 (Δη_F-actin+Tpm1.7_). As can be seen from Figure 6b, while Tpm1.7 E40K protein exhibited very similar to WT effects, the Tpm1.7 E54K have shown better protection of F-actin from cof-1.

### 2.5. Actomyosin Interactions in the Presence of Tpm1.7 WT and Its E40K and E54K Variants

Actomyosin interactions in non-muscle cells depend on Tpm isoforms. The Tpm-mediated inhibition of myosin interaction with actin has been described for Tpm1.7 and myosin Myo1C [32]. In the present study, we estimated the effect of Tpm1.7 isoform on actomyosin interactions using an in vitro motility assay with skeletal myosin (Table 4). Tpm1.7 WT reduced the sliding velocity of F-actin by half. The Tpm1.7 E40K mutant reduced the sliding velocity of F-actin-Tpm filaments by 1.4-fold compared to that with the WT protein. Interestingly, the sliding velocity of filaments with Tpm1.7 E54K did not differ from that of filaments of pure F-actin.

## 3. Discussion

Mutations in the *TPM1* gene are well-known to underlie the development of cardiomyopathies. This gene encodes many isoforms, so a single mutation can result in the expression of several different proteins carrying an a.a. substitution. The Tpm1.7 isoform is related to early development (the cleavage furrow), neuronal cells and stress fibers, and significantly determines the properties of actin filaments. In this study, we investigated the effects of cardiomyopathy-causing mutations E40K and E54K in the human *TPM1* gene on the non-muscle cytoplasmic tropomyosin isoform Tpm1.7.

The properties of cardiac sarcomere Tpm1.1 isoform carrying these mutations have been previously well studied, and here we compared the effects of the E40K and E54K substitutions on the Tpm1.1 and Tpm1.7 isoforms.

### 3.1. The Effects of the E40K and E54K Mutations on the Structure and Properties of Tpm1.1 and Tpm1.7

The E40K and E54K mutations are located at position *e* of the heptad repeats, which participate in additional stabilization of the coiled-coil structure through electrostatic interactions with a.a. at position *g* of the other coil. The E (Glu) to K (Lys) replacement causes the appearance of an opposite a.a. residue charge.

Thermal stability assays performed used to determine the structural changes caused by these mutations. The CD and DSC data were in a good agreement (Figure 2 and Figure 3) and showed significant effect of the studied mutations on the thermal stability of the Tpm1.7 molecule. The E40K and E54K mutations resulted in destabilization of calorimetric domains 2 of Tpm1.7, which corresponds to the C-termini of the molecule (−2.3 °C for E40K and −3.4 °C for E54K). They also cause a decrease of T_m_ for calorimetric domain 3, which reflects melting of the N terminus (−3.8 °C for E40K, and −0.9 °C for E54K). The E54K mutation also affected the first thermal transition (−2.7 °C), and resulted in the appearance of a new thermal transition at 55.2 °C. Similar effects were previously observed for the Tpm1.1 isoform with these mutations [8]. The E40K mutation in Tpm1.1 also resulted in the destabilization of the N-terminal domain, due to the destruction of the Glu40 to Arg35 inter-strand salt bridge. The effects of the E54K substitution on N-terminal part are also very similar for both Tpm isoforms. The Glu54 may form inter-strand interactions with Lys51 in position *b* and Lys49 in position *g* [33]; however, is more likely part of the actin-binding site [34]. The E54K substitution in Tpm1.1 resulted in destabilization of the N-terminal domain and cause addition of a new high-temperature domain that unfolded at 55.2 °C [8]. However, there are also significant differences between mutated Tpm1.1 and Tpm1.7 isoforms. For Tpm1.1, all changes in its thermal stability affected exclusively the N-terminal part of molecules, while domains 1 and 2 are presented the same as for Tpm1.1 WT. In contrast, the studied mutations in the Tpm1.7 molecule cause effects over longer distances and affect domains 1 and 2 in addition to domain 3.

Due to head-to-tail interactions, Tpm1.7 demonstrated one of the highest viscosities among all Tpm isoforms [12]. Both Tpm1.7 E40K and E54K solutions had viscosity similar to the viscosity of Tpm1.7 WT (Table 2). This indicates that head-to-tail interactions of the adjacent molecules remain stable. The E40K and E54K substitutions did not affect affinity of Tpm1.7 to F-actin (Figure 3). The external glutamates at positions 40 and 54 were postulated by “consensus” to be actin binding residue [35]. For Tpm1.6, the E54 residue was predicted to form salt bridge with K328 of actin [34]. In Tpm1.1, both E40K and E54K mutations reduced the affinity of Tpm for F-actin, but in different states (in “On” and “Off” states for E40K and E54K, respectively) [8]. However, here we did not observe any critical changes in the Tpm1.7 affinity for F-actin due to these point mutations.

The bending stiffness of F-actin/Tpm1.7 is almost the same as for F-actin/Tpm1.9 [36]. Such effects may occur due to the properties of the Tpm1.7 and Tpm1.9 isoforms (the exon composition differs only in the N-terminal part: 1a2b in Tpm1.7 and 1b in Tpm1.9). Both isoforms also display strong end-to-end interactions. It is likely that Tpm1.7 is required to form actin filaments of a high rigidity, such as stress fibers. In contrast, stiffness of F-actin/Tpm1.7 E40K filaments is lower than F-actin/Tpm1.7 WT filaments. Bending stiffness of F-actin/Tpm1.7 E54K is similar to that of F-actin/Tpm1.7 WT filaments, while it is lower for F-actin/Tpm1.7 E40K (Table 3). Since the Tpm1.7 E40K affinity for actin and end-to-end interactions are very similar to those of the wild-type protein, we speculate that this might correlate with the destabilization of Tpm molecule by the E40K mutation (Table 1).

### 3.2. The Effects of Tpm1.7 E40K and E54K Variants on Actin-Myosin Interplay

Various myosins exhibit different interactions with Tpm-covered actin filaments. In previous studies it has been shown that in vitro Tpm1.7 inhibits the motility of actin filaments driven by non-muscle myosin (Myo1C), and resists Myo1C-induced dislocation from F-actin [32]. Tpm1.7 also inhibits interactions between F-actin and mitochondrial myosin Myo19 [37]. Similar effects were observed for non-muscle myosin IIa (NMIIa) (the Tpm1.7 slightly reduced myosin NMIIa ATPase compared to bare actin) [28]. We applied the in vitro motility assay with skeletal myosin and tested the effects of Tpm1.7 WT or its mutant variants on the sliding velocities of actin filaments (Table 4). Tpm1.7 WT alone reduced by half a sliding velocity of actin filaments, similar to its effects on Myo1C, NMIIa, and Myo19 motors. The E40K mutant reduced the sliding velocity of F-actin by 3-fold more than the wild-type protein, while the velocity of actin decorated with Tpm1.7 E54K variant did not differ from that of bare F-actin (Table 4). The Mirza et al. found that the F-actin coated with Tpm1.1 E40K showed an 18% lower myosin S1 ATPase activity compared to the filaments with Tpm1.1 WT. However, filaments of F-actin-Tpm1.1 E54K exhibited the maximal sliding velocity of thin filaments at saturating calcium concentration that was slightly higher than for Tpm1.1 WT [8]. Since our results for Tpm1.7 isoform well correlate with data obtained for Tpm1.1, it can be explained by effects of mutations, either on position of Tpm on actin filament or its resistance to myosin-induced dislocation.

### 3.3. Mutant Variants of Tpm1.7 E40K and E54K Affect Cofilin-1 Activity

The dynamics of the actin filaments is regulated by members of ADF/cofilin family, which include ADF and cofilins 1 (cof-1) and cofilin-2 (cof-2). Tpm and cofilins mutually influence their binding to the actin filament surface.

Tpm1.7 is known to protect against cof-1-induced actin severing [28]. We also previously confirmed the ability of Tpm1.7 to inhibit cof-1 depolymerization activity using viscosity measurements. [29]. Here, the interplay of Tpm1.7 E40K and Tpm1.7 E54K with cof-1 was studied by measuring the length of actin filaments after incubation with cof-1. The isoforms obtained from *TPM1* gene (Tpm1.1, 1.6, 1.7, 1.8, and 1.9) display similar features for cofilin. It is known from Actin/Tpm1.6/cof-1 models that ADF/cofilin and Tpm1.6 compete for actin binding sites, as supported by experimental data [34]. All Tpm1.7 variants significantly elongated actin filaments (Figure 5c,e,g) that correlated with the viscosity of solutions (Figure 6). Tpm1.7 protected actin filaments from cof-1-induced severing (Figure 5d,f,h). The filaments length distribution of F-actin/Tpm1.7 WT with cof-1 was more similar to that of F-actin/Tpm1.7 (Figure 5c,d). Both Tpm1.7 E40K and Tpm1.7 E54K showed the protective action against F-actin severing activity of cof-1 (Figure 5g,h). However, Tpm1.7 E54K exhibited slightly higher protection against cof-1 activity compared with Tpm1.7 WT and Tpm1.7 E40K (Figure 5 and Figure 6). Previously, it was shown for Tpm3.12 isoform that a single a.a. substitution could impair the properties of Tpm towards cofilin. Tpm3.12 R91C inhibited actin severing by cof-2 more strongly than Tpm3.12 WT [38].

Considering that Tpm1.7 E54K affected both myosin interaction with F-actin filaments and cofilin-induced severing (Table 3, Figure 6), and the fact that cof-1 and myosin share the same interaction sites on the F-actin surface [39,40,41], it can be assumed that Tpm1.7 E54K altered the accessibility of this site on the actin surface.

## 4. Materials and Methods

### 4.1. Protein Preparations

Human recombinant Tpm1.7 proteins and its mutants contained Ala-Ser extension on N-termini to mimic naturally occurring N-terminal acetylation of Tpm [42]. The E40K and E54K mutants were obtained by Q5 site-directed mutagenesis kit (NEB, New England BioLabs, Ipswich, MA, USA). The following oligonucleotides were used:

5′- CAGCTGAAGGATGAGCTGGTGTCAC -3′ as the forward, and 5′- CTTGCTCCTGTCTTCCGCCGC -3′ as the reverse primers for E40K mutant, and 5′- GGCACCAAGGATGAACTGGACAAATAC -3′ as the forward, and 5′- CTTGAGTTTCTTTTGCAGTGACACCAGCT -3′ as the reverse primers for E54K mutant. The resulting CDS was verified by sequencing. Tpms were expressed in *E. coli* strain C41 and purified by subsequent heating at 85 °C during 6 min, isoelectric precipitation, and by anion-exchange chromatography on a lexCap Smac Q 40 column (Smart-Lifesciences, Changzhou, China) [43]. The Tpm concentrations were determined spectrophotometrically at 280 nm using an *E*^1%^ of 1.8 cm^−1^.

Actin was extracted from *m. psoas* of a rabbit and purified using standard methods [44]. The G-actin concentration was determined spectrophotometrically at 290 nm using an *E*^1%^ of 6.3 cm^−1^. Filamentous actin (F-actin) was polymerized from monomeric G-actin at the concentration of 2 mg/mL by addition of 2 mM MgCl_2_ and 100 mM KCl.

Recombinant human cofilin-1 (cof-1) was obtained as described previously [45] in the *E. coli* strain C41. The IPTG was added to a final concentration 1 mM, and the cells were harvested after an overnight expression at 30 °C. The cells were lysed by sonication in a 50 mM HEPES-Na buffer at pH 7.3, containing 300 mM NaCl and 20 mM imidazole. The cof-1 was purified using metal affinity chromatography on a HisCap 6FF 5 mL (Smart-Lifesciences, Changzhou, China) column. The purified protein was dialyzed and stored in a 50 mM HEPES-Na buffer at pH 7.3, containing 150 mM NaCl, 0.1 mM PMSF, and 14 mM β-ME. The purity of the cofilin-1 preparations was no less than 98%. The protein concentration was determined spectrophotometrically using an *E*^1%^ value of 7.8 cm^−1^ at 280 nm.

Myosin for F-actin immobilization was extracted from *m. psoas* of a rabbit by a standard protocol [46].

### 4.2. Circular Dichroism (CD)

Far-UV CD spectra of Tpm1.7 samples were recorded at 5 °C on a Chirascan CD spectrometer (Applied Photophysics, Surrey, UK) in 0.02 cm cells at the protein concentration 1.0 mg/mL. The thermal unfolding in a temperature range of 5 °C to 75 °C was detected by measuring the molar ellipticity of samples at 222 nm (θ_222_) at a constant heating rate of 1 °C/min. All experiments were performed in a 30 mM HEPES-Na buffer, pH 7.3, containing 100 mM NaCl. To check the reversibility of Tpm denaturation, the samples were subjected to subsequent cycles of heating–cooling, followed by CD spectra measurements. The first derivative analysis was applied to determine the temperature of the maximum (T_m_) of the thermal transitions. Two measurements were performed for each Tpm sample.

### 4.3. Differential Scanning Calorimetry (DSC)

DSC experiments were conducted as described earlier on a MicroCal VP-Capillary DSC differential scanning calorimeter (Malvern Instruments, Northampton, MA, USA) at a heating rate of 1 °C/min and a protein concentration of 2 mg/mL in 30 mM HEPES-Na buffer, pH 7.3, containing 100 mM NaCl. The thermal unfolding of Tpm was fully reversible. The temperature dependence of the excess heat capacity (∆Cp) was analyzed and plotted using Origin software (version 7, MicroCal Inc., Northampton, MA, USA). Deconvolution analysis of the heat sorption curves, i.e., their decomposition into separate thermal transitions (calorimetric domains), was based on the model described in [47]. The following parameters were obtained for each calorimetric domain: the ∆H_cal_, and T_m_, which marks the midpoint of the respective thermal transition. The measurements were repeated twice.

### 4.4. Viscosity Measurements

The viscosity measurements of the Tpm solutions were performed using a falling ball microviscometer Anton Paar AMVn (GmbH, Graz, Austria) in a 0.5 mL capillary at 20 °C as previously described. All measurements were performed in a 30 mM HEPES-Na buffer, pH 7.3, containing 100 mM NaCl. The viscosity of each Tpm sample was measured at least six times, and the viscosity values after subtracting the buffer viscosity (Δη) were averaged.

### 4.5. Co-Sedimentation of Tpm Species with F-Actin

The apparent affinity of Tpm1.7 and its mutants for F-actin was estimated using a co-sedimentation assay [12]. Briefly, 10 µM F-actin was mixed with increasing concentrations of Tpm (0.5, 1.5, 3, 4.5, 6, and 7.5 µM) at 20 °C in 30 mM HEPES-Na buffer, pH 7.3, containing 200 mM NaCl. F-actin was pelleted by ultracentrifugation at 100,000 *g* for 40 min, and equivalent samples of the pellet and the supernatant were subjected to SDS-PAGE. Protein bands were scanned and analyzed using ImageJ 1.53k software (Scion Corp., Frederick, MD, USA). Three measurements were performed for each Tpm sample.

### 4.6. Bending Stiffness of F-Actin Decorated by Tpm

The bending stiffness of F-actin or actin–Tpm filaments was estimated by using an optical trap as described previously [48]. In brief, actin filaments with and without Tpm1.7 variants were linked to two microbeads held by a two-beam optical trap. The filament was stretched with an acousto-optically controlled trap, pulling the motor bead in 50 nm steps, while the other bead was used as a force transducer. The microphotographs showing bead distances were taken after each step and were used to plot the force–distance curve. The experimental cell was filled by F-actin or Tpm/F-actin complexes at a molar ratio of 10:1 in a buffer solution containing 25 mM KCl, 25 mM imidazole, 4 mM MgCl_2_, 1 mM EGTA, and 20 mM DTT, pH 7.5. Scavenger system with 0.2 mg/mL glucose oxidase, 0.05 mg/mL catalase, and 3 mg/mL glucose was also added to the solution.

### 4.7. Cofilin Activity Assay

To determine the severing/depolymerizing activity of cof-1 on F-actin filaments with Tpm1.7 WT and its mutants, we applied fluorescence microscopy and viscosity measurements. 10 µM F-actin and 3.3 µM Tpms were mixed in a 30 mM HEPES-Na buffer at pH 7.5, containing 100 mM NaCl and 1 mM DTT. After 20 min of incubation, cofilin-1 was added to the samples to a final concentration of 1 µM, and the length of filaments or viscosity of solutions were measured 60 min after its addition. All experiments were performed at 20 °C. The obtained F-actin filaments were stained 10 min with TRITC-phalloidin (Sigma Chemical Co., St. Louis, MO, USA) and loaded into an experimental flow cell with skeletal myosin immobilized on the nitrocellulose-coated inner surface.

For immobilization, 300 µg/mL myosin in an AB buffer (25 mM KCl, 25 mM imidazole, 4 mM MgCl_2_, 1 mM EGTA, and 20 mM DTT, pH 7.5) containing 0.5 M KCl was loaded into the experimental flow cell. After 2 min, 0.5 μg/mL BSA was added for 1 min. The F-actin samples were diluted by 200 times with a 30 mM HEPES-Na buffer, pH 7.5, containing 100 mM NaCl and 1 mM DTT, and an oxygen scavenger system (3.5 mg/mL glucose, 20 µg/mL catalase, and 0.15 mg/mL glucose oxidase). Fluorescently labeled F-actin was visualized with an Axiovert 200 (Carl Zeiss, Oberkochen, Germany) inverted epifluorescence microscope equipped with a 100 9/1.45 oil-immersion alpha Plan-Fluar objective and an EMCCD iXon-897BV (Andor Technology, Belfast, UK) video camera, with 25–30 fields recorded for each sample.

The lengths of F-actin filaments (1000 filaments for each sample) were analyzed using ImageJ 1.53c software (Scion, Frederick, MD, USA). A two-sample Kolmogorov–Smirnov test for pairwise comparison (with Bonferroni correction) was applied to determine whether there were significant differences.

The viscosity of F-actin/Tpm samples containing cof-1 was measured as described in Section 4.4 and [45].

### 4.8. In Vitro Motility Assay

The motility chamber with skeletal myosin was prepared as described in Section 4.7. Unattached myosin was subsequently washed out, and 50 µg/mL of non-labeled F-actin in a AB buffer containing 25 mM KCl, 25 mM imidazole, 4 mM MgCl_2_, 1 mM EGTA, 20 mM DTT, and 2 mM ATP, pH 7.5, was added for 5 min to block nonfunctional myosin molecules. Then a mixture of 10 nM TRITC-phalloidin labeled F-actin and 100 nM of Tpm in an AB buffer with 2 mM ATP and oxygen scavenger system (3.5 mg/mL glucose, 20 µg/mL catalase, and 0.15 mg/mL glucose oxidase) was uploaded into the cell. Measurements of the sliding velocities of TRITC-phalloidin–labeled F-actin decorated with Tpm1.7 variants were performed at 30 °C in three independent experiments. The sliding velocity was estimated by GMimPro2023 software [49], according to criteria previously described [50]. Comparisons were performed by paired *t*-test or Mann–Whitney *U*-test at a significance level of 0.05 (*p* < 0.05).

## 5. Conclusions

Due to alternative splicing, the human *TPM1* gene expresses ten Tpm isoforms. For that reason, the missense mutations described for this gene might affect several isoforms of Tpm. Cardiomyopathy-associated mutations E40K and E54K are located in exon 2b of the *TPM1* gene and may be in all Tpm isoforms containing this exon. We became interested in whether these mutations could affect the cytoplasmic Tpm1.7 isoform, which is not related to muscles, but plays an important role in development of neuronal tissue, cell migration and formation of stress fibers. Indeed, we found that the E40K and E54K mutation altered the structural properties and stability of the Tpm1.7 molecule, yet did not change Tpm longitudinal assembly or the Tpm1.7 affinity for F-actin. However, they influenced properties of F-actin filaments and its interaction with other actin-binding proteins. For example, the E40K mutation decreased bending stiffness of F-actin filaments. Results obtained in the in vitro motility assay indicate that both mutations affected the interaction of F-actin-Tpm1.7 filaments with myosin. The E54K mutation led to a Tpm1.7 variant that protected actin filaments from cofilin-induced severing better than Tpm1.7 WT. Summarizing our data, we can conclude that cardiomyopathic mutations in the *TPM1* gene affect not only the sarcomeric isoform Tpm1.1, but can also alter the properties of non-muscle Tpm isoforms, suggesting a potential pathogenesis mechanism unrelated to the contractile apparatus. This fact should be taken into account when studying the mechanisms of pathogenesis of cardiomyopathies associated with mutations.

## Figures and Tables

**Table 1:** Calorimetric parameters obtained from the DSC data for individual thermal transitions (calorimetric domains) of Tpm1.7 WT and Tpm1.7 with mutations E40K and E54K.

Protein	Domain	T_m_^#^ (°C)	ΔH_cal_^§^ (kJ mol^−1^)	ΔH_cal_ (% of Total ΔH_cal_)	Total ΔH_cal_ (kJ mol^−1^)
Tpm1.7 WT	Domain 1	35.2	145	11	1345
	Domain 2	44.2	755	56	
	Domain 3	51.4	445	33	
Tpm1.7 E40K	Domain 1	35.5	88	9	952
	Domain 2	41.9	483	51	
	Domain 3	47.6	382	40	
Tpm1.7 E54K	Domain 1	32.8	92	10	853
	Domain 2	40.8	405	49	
	Domain 3	50.5	282	33	
	Domain 4	55.2	73	8	

**^#^** The error of the transition temperature (T_m_) did not exceed ±0.2 °C. ^§^ The relative error of the given values of the calorimetric enthalpy (ΔH_cal_) did not exceed ±10%.

**Table 2:** Excess solution viscosity of Tpm1.7 WT, Tpm1.7 E40K, and Tpm1.7 E54K variants.

Sample	Excess Viscosity Over the Buffer (mPa · s)	
	1 mg/mL	0.5 mg/mL
Tpm1.7 WT	1.273 ± 0.031	0.383 ± 0.003
Tpm1.7 E40K	1.337 ± 0.013 *	0.443 ± 0.004 *
Tpm1.7 E54K	1.193 ± 0.006 *	0.405 ± 0.004 *

The symbol * indicates a statistically significant difference in the excess solution viscosity of Tpm1.7 E40K and Tpm1.7 E54K from the excess solution viscosity of Tpm1.7 WT, Student *t*-test, *p* < 0.05.

**Table 3:** Bending stiffness of F-actin filaments decorated with Tpm1.7 WT, Tpm1.7 E40K or Tpm1.7 E54K mutants.

Sample	Bending Stiffness, K × 10^26^ N·m^2^		
	Median	Interquartile Range	N
F-actin	2.9	2.15–3.6	25
F-actin + Tpm1.7 WT	4.7 *	3.5–7.25	17
F-actin + Tpm1.7 E40K	3.5 *^#^	2.55–4.55	21
F-actin + Tpm1.7 E54K	4.2 *	3.8–4.9	19

^#^—statistically significant difference between the bending stiffness of the filaments with Tpm1.7 mutant and Tpm1.7 WT. *—statistically significant difference between the stiffness of all F-actin filaments decorated with Tpm1.7 filaments and F-actin. Statistical differences were assessed using the nonparametric Mann–Whitney U test at a significance level of *p* < 0.05.

**Table 4:** Sliding velocities of F-actin filaments alone, with Tpm1.7 WT, or its E40K and E54K mutants.

	Sliding Velocities (µm/s)		
F-actin	+Tpm1.7 WT	+Tpm1.7 E40K	+Tpm1.7 E54K
4.3 ± 0.3	2.2 ± 0.3 ^#^	1.5 ± 0.2 *^#^	4.1 ± 0.5 *

The data presented as mean ± SD. The three independent experiments were performed for each sample; the sliding velocities of no less than ten filaments were estimated. The symbol * indicates a statistically significant difference in the sliding velocity of F-actin-Tpm filaments in the presence of tropomyosin with mutations from the velocity of filaments with WT Tpm1.7, Mann’s U test, *p* < 0.05. The symbol ^#^ indicates a statistically significant difference in the sliding velocity of F-actin-Tpm filaments from the velocity of bare F-actin, Mann’s U test, *p* < 0.05.

## Data Availability

All data are presented in the article.

## Abbreviations

The following abbreviations are used in this manuscript:

Tpm	Tropomyosin
Cof-1	Cofilin-1
F-actin	Filamentous actin
CD	Circular dichroism
DSC	Differential scanning calorimetry
